# The initial results of MRI-TRUS fusion prostate biopsy in high volume tertiary center

**DOI:** 10.2478/raon-2024-0060

**Published:** 2024-11-28

**Authors:** Tomaz Smrkolj, Milena Taskovska, Iztok Ditz, Klemen Cernelc, Simon Hawlina

**Affiliations:** Department of Urology, Ljubljana University Medical Centre, Ljubljana, Slovenia; Chair of Surgery, Faculty of Medicine, University of Ljubljana, Ljubljana, Slovenia

**Keywords:** prostate cancer, targeted prostate biopsy, learning curve, complications

## Abstract

**Background:**

Multiparametric magnetic resonance imaging (mpMRI) is a prerequisite for targeted prostate biopsy. The aim of our study was to evaluate the performance and learning curve of the mpMRI-transrectal ultrasound (TRUS) software image fusion (MRI-TRUS fusion) biopsy (BX) process in the first year after its introduction in our urology department.

**Patients and methods:**

MRI-TRUS fusion BX was performed in 293 patients with at least one Prostate Imaging-Reporting and Data System (PIRADS) ≥3 lesion. The proportion of patients and lesions with positive histopathologic result for prostate cancer (PCa) was analyzed. The learning curve for MRI-TRUS fusion BX was assessed at institutional and individual level. Positive BX lesions were further analyzed by PIRADS and Gleason scores.

**Results:**

The proportion of patients with positive histopathologic results for targeted BX, systematic BX, and combined BX was 53.9%, 47.9%, and 63.5%, respectively. The chi-square test for the proportion of PCa positive patients showed no significant difference between the time-based patient groups at the institutional level and no significant difference between individual urologists. PIRADS score (p < 0.001), total PSA concentration (p = 0.05), prostate volume (p < 0.001) and number of cores per lesion (p = 0.034) were significant predictors of a positive histopathologic result in a lesion-based analysis. Clinically significant PCa (csPCa) was confirmed in 34.7% of the 412 BX lesions and 76.4% of the 187 positive PCa lesions.

**Conclusions:**

MRI-TRUS fusion targeted BX significantly improves the overall rate of PCa detection compared with systematic BX alone. No steep learning curve was observed in our urologists. The proportion of lesions with clinically insignificant PCa was low, limiting overdiagnosis of PCa.

## Introduction

Prostate cancer (PCa) is the second most common cancer in men worldwide. The estimated age-standardized incidence rate is highest in Western and Northern Europe, North America, Australia and New Zealand.^1^ In Slovenia, PCa is the most common solid neoplasm in men (excluding skin cancer) with a share of 18.6% and an estimated incidence rate of 162 per 100,000 in 2022.^2^ The incidence rate of PC in Western countries and Slovenia has increased dramatically from the early 1990s to the last decade, mainly due to the use of prostate specific antigen (PSA) as a tumor marker for PCa.^2,3,4^

PSA is a serine protease that is produced almost exclusively by epithelial cells in the prostate. Therefore, serum PSA is an organ specific marker that can be elevated in benign prostate diseases such as inflammation and prostate enlargement in addition to PCa.^5,6^ The majority of men with elevated PSA levels and/or suspicious digitorectal examination (DRE) of prostate underwent transrectal (systematic) template biopsy (BX) in the first decades after the introduction of the PSA to confirm the PCa diagnosis and initiate treatment. Systematic BX templates have evolved over the years in terms of the number and location of cores^7,8^, however, these templates have focused primarily on the posterior and lateral peripheral zones and much less on the transitonal zone and anterior portion of the prostate.

The natural history of PCa in many older men is protracted and does not cause significant health problems during their expected lifespan. The concept of incidentally found and clinically insignificant PCa (cisPCa) was introduced to reduce the risk of overtreatment.^3,9^

To limit the number of unnecessary BXs in men with elevated PSA due to benign disease or cisPCa, novel tumor markers have been developed that may aid in the decision to perform BX, e.g., pro-PSA, Prostate Health Index, prostate cancer antigen 3 (PCA3), Select MDX^10,11,12,13^, however, tumor markers do not provide information about the location of PCa within the prostate.

In the last ten years, multiparametric magnetic resonance imaging (mpMRI) has become the imaging modality of choice for the diagnosis of PCa.^14^ mpMRI has an excellent sensitivity of 91% to 95% for clinically significant PCa (csPCa) and a low yield (21% to 29%) for small cisPCa, while having a high negative predictive value (NPV) of 63% to 98%.^15,16,17^ mpMRI reporting has been standardized in the Prostate Imaging-Reporting and Data System (PIRADS) and each lesion is classified into one of 5 groups of increasing risk for csPCa according to radiological criteria.^18^

Another major advantage of mpMRI is that it provides information about the exact location of the suspicious lesion in the prostate. In the pre-mpMRI era, suspicious hipoehoic lesions in the prostate were identified with transrectal ultrasound (TRUS) in about half of patients with PCa.^19,20^ With mpMRI, it is possible to identify suspicious lesions on the BX under cognitive guidance, TRUS – mpMRI software image fusion (MRI-TRUS fusion) or inbore MRI BX. Although some studies found no significant difference between the performance of the three BX targeting methods mentioned above^21,22^, other studies report advantages of MRI-TRUS fusion and in-bore MRI BX compared to cognitive guidance.^23,24^

The aim of our study was to evaluate the performance and learning curve of the MRI-TRUS fusion BX process in the first year after its introduction in a high-volume clinical setting.

## Patients and methods

### Patients

The study was approved by the National Medical Ethics Committee of the Republic of Slovenia (0120-69/2023/3) and was conducted in full compliance with the principles of the Declaration of Helsinki.

Patients with at least one clearly defined PIRADS ≥ 3 lesion who underwent MRI-TRUS fusion targeted BX in an outpatient clinic of the Urology Department of UMC Ljubljana were included in the study. The exclusion criteria were: no clearly defined suspicious lesion on mpMRI of the prostate, patients in whom only systematic BX was performed, patients with a contraindication for transrectal ultrasound (TRUS) BX, in whom biopsy was cancelled.

A total of 293 patients who underwent MRITRUS fusion targeted BX between June 2021 and June 2022 were retrospectively included in our study.

### Detection methods

#### Multiparametric MRI

In patients scheduled for MRI-TRUS fusion targeted BX, mpMRI was performed by different radiologists on different MRI machines in several public hospitals and several private centers in the Republic of Slovenia. The choice of center for mpMRI was at the discretion of the patients.

#### Contouring of MRI lesions

Contouring of prostate boundaries and each suspicious lesion was performed using MIM software (version 7.1.2, MIM software inc., Cleveland, OH, USA) by 3 certified urologists (with 19, 7 and 6.5 years of experience on systematic BX) who had previously participated in several certified mpMRI reading courses. Each radiologist-reported lesion with PIRADS ≥ 3 was identified and contoured on the T2 axial, T2 sagital, DWI and ADC mpMRI maps. In a minority of cases, T1 contrast-enhanced axial mpMRI maps had to be reviewed to clearly identify the lesion. PIRADS score of a lesion and its largest diameter were recorded for further analysis.

#### Transrectal MRI fusion targeted BX

All patients received peroral antibiotic prophylaxis with phosphomycin (3g) the evening before BX. No bowel preparation or swab sampling was performed before the procedure. MRI-TRUS fusion targeted BX was performed on a bk3000 ultrasound machine (BK Medical Holding Company, Inc., Peabody, Massachusetts, USA) equipped with freehand 3D electromagnetic tracking device (Ascension Technology Corporation, Shelburne, VT, USA) with MIM software (version 6.9.7, MIM software inc., Cleveland, OH, USA) installed. A Triplane 12 MHz transrectal transducer was used in all patients. Prior to BX, a periprostatic anesthetic block with 6 ml of 2% lidocaine was administered bilaterally at the base of the prostate through the BX needle sheath. During the BX session, DRE of the prostate was performed and the volume of the prostate was measured by TRUS examination. During targeted BX, up to three contoured lesions were sampled from each patient. Systematic BX was then performed on the remaining, non-sampled lateral peripheral zone of the prostate. The number of systematic BX cores depended on the number and position of the targeted BX cores and prostate volume. Post-BX complications were determined via the hospital information system by reviewing patient records within the 1-month post-biopsy period.

The number of certified urologists and residents performing MRI-TRUS fusion targeted BX gradually increased over the 1-year period. Each new member of the BX team was instructed in image fusion and the proper technique of targeted BX and was supervised by one of the three certified urologists of the contouring team for at least 5 to 10 patients.

#### Histopathology report

The BX cores of the patients were analyzed in the histopathology laboratory of the Institute of Pathology at the Faculty of Medicine in Ljubljana, Slovenia. The histopathologic results were divided into two categories: *the negative group* (BPH, prostatitis and high-grade prostatic intraepithelial neoplasia (HGPIN)) and *the positive - malignant group* (PCa, atypical small acinar proliferation (ASAP) and suspected PCa). Gleason grade and score of each targeted lesion and systemic biopsy cores were noted separately for analysis. We defined csPCa as a Gleason score ≥ 7 and a Gleason grade group ≥ 2.

### Statistical analysis

Data were analyzed using SPSS software (Statistical Package for the Social Sciences, version 29.0, IBM Corp., Armonk, NY, USA). Mean, median, minimum value, maximum value and standard deviation were used to indicate numerical variables. The proportion of positive histopathologic results was calculated for both patient-based and lesion-based analysis. Pearson’s chi-square test was used to analyze the effects of categorical variables on the proportion of patients with positive histopathological findings. Univariate binary logistic regression was performed to analyze the impact of numeric variables on the proportion of patients with positive histopathology. In the lesion-based analysis, a multiple binary logistic regression analysis was performed for numeric and categorical covariates affecting a positive histopathologic result. The proportion of post-procedural complications was calculated. A p-value of less than 0.05 was considered statistically significant.

## Results

In the first year, we performed MRI-TRUS fusion targeted BX in 293 patients, while systematic BX was performed in 288 of these patients. In 25 patients 3 targeted lesions were sampled, in 69 patients 2 lesions were sampled and in the remaining 199 patients 1 lesion was sampled (Table 1).

**TABLE 1.** Patient and lesion characteristics

	**min**	**max**	**mean ± SD**	**median**
Age (years)	35	91	69.0 ± 7.9	70.0
*Total PSA concentration (ng/mL)	0.45	115.4	9.7 ± 11.7	7.0
Prostate volume (ml)	15	313	52.8 ± 32.3	45
No. lesions biopsied in a patient	1	3	1.4 ± 0.6	1.0
Largest diameter of lesion (mm)	3.0	41.0	12.8 ± 6.6	11.5
No. of cores per lesion targeted BX	1	8	4.26 ± 1.2	4.0
No. of cores systematic BX	0	14	6.9 ± 1.8	6.0
No. of MRI-TRUS fusion targeted BX performed by urologist before	1	65	22.5 ± 19.7	20.0

* PSA = in patients with 5-alpha reductase inhibitors (5ARI) therapy total PSA concentration was doubled. Number of patients is 293, number of lesions is 412.

BX = biopsy; MRI-TRUS = magnetic resonance imaging - transrectal ultrasound; SD = standard deviation

### Patient based analysis

The proportion of patients with a positive histopathologic result for targeted BX, where at least one of the targeted BX was positive, was 53.9% (158/293). The proportion of patients with a positive histopathology result for systematic BX was 47.9% (138/288). The overall proportion of patients with positive histopathology for at least one targeted or systematic BX was 63.5% (186/288). 28 patients with negative targeted BX had positive systematic BX, and in this group histopathology reports had identified 3 patients with ASAP, 10 patients with G6 (3+3), 9 patients with G7 (3+4), 3 patients with G7 (4+3), 2 patients with G8 (4+4) and one patient with G9 (4+5). Therefore, the targeted BX alone would have missed 5.2% (15/288) csPCa in all patients who had targeted BX and added 4.5% (13/288) cisPCa.

To assess the impact of the learning curve on the proportion of patients with a positive histopathologic result for targeted BX, systematic BX, and overall BX (targeted and systematic BX combined) at the institutional level, a chi-square test for independence was performed. Based on the date of BX, patients were divided into two, three and six time-based groups (Table 2). No significant differences were found.

**TABLE 2.** Comparison of time-based patient groups for learning curve estimation on institution level

	**Targeted BX**			**Systematic BX**			**Overall BX**		
	Pearson χ^2^	df	sig	Pearson χ^2^	df	sig	Pearson χ^2^	df	sig
2 patient groups	0.458	1	0.498	0.658	1	0.417	0.291	1	0.590
3 patient groups	2.145	2	0.342	2.265	2	0.322	1.373	2	0.503
6 patient groups	4.807	5	0.440	2.872	5	0.720	2.322	5	0.803

BX = biopsy; df = degrees of freedom; sig = significance

A chi-square test of independence was performed to assess the effect of the learning curve on the proportion of patients with a positive histopathologic result for targeted BX, systematic BX, and overall BX at the individual urologist level. For targeted BX, Pearson χ^2^, df and sig were 9.124, 12 and 0.692, respectively. For systematic BX Pearson χ^2^, df and sig were 10.431, 12 and 0.578, respectively. For overall BX Pearson χ^2^, df and sig were 9.465, 12 and 0.613, respectively. In addition, univariate binary logistic regression analysis of the effect of the number of previous MRI-TRUS fusion targeted BX sessions performed by a urologist on the probability of a positive histopathologic result at subsequent BX yielded odds ratios of 1.011 (p = 0.169), 1.007 (0.372), and 1.014 (p = 0.107) for targeted BX, systematic BX, and overall BX (targeted and systematic combined), respectively. No significant differences were found.

### Lesion based analysis

Of 412 targeted BX lesions, 187 were positive for PCa (45.4%). 127 lesions were classified as PIRADS 3 (30.8%), 204 lesions as PIRADS 4 (49.5%) and 81 lesions as PIRADS 5 (19.7%). The proportion of positive targeted BX lesions increased with increasing PIRADS score (Table 3).

**TABLE 3.** Proportion of positive targeted biopsy (BX) lesions categorised by Prostate Imaging-Reporting and Data System (PIRADS) score

	**Negative BX**	**Positive BX**	**Total**
PIRADS 3 (%)	104 (81.9%)	**23 (18.1%)**	127
PIRADS 4 (%)	108 (52.9%)	**96 (47.1%)**	204
PIRADS 5 (%)	13 (16.0%)	**68 (84.0%)**	81

Multiple binary logistic regression analysis on the covariates influencing a positive histopathologic result in targeted BX of a lesion revealed significant odds ratios for prostate volume, total PSA concentration, PIRADS score, and number of cores per sampled lesion (Table 4).

**TABLE 4.** Results of multiple logistic regression of positive histopathologic result

	**Sig**	**Odds ratio**	**95% C.I. for odds ratio**	
			**Lower**	**Upper**
Max lesion diameter	0.945	0.998	0.955	1.044
**Prostate volume**	**< 0.001**	**0.978**	**0.968**	**0.988**
*Total PSA concentration	**0.050**	**1.050**	**1.000**	**1.103**
**PIRADS 3**	**< 0.001**			
**PIRADS 4**	**< 0.001**	**3.208**	**1.848**	**5.567**
**PIRADS 5**	**< 0.001**	**16.222**	**6.475**	**40.645**
**Number of cores per lesion**	**0.034**	**1.238**	**1.016**	**1.508**
Number of previous patients with targeted BX performed by urologist	0.113	1.013	0.997	1.028

* PSA = in patients with 5-alpha reductase inhibitors (5ARI) therapy PSA concentration was doubled.

BX = biopsy; C.I. = confidence interval; PIRADS = Prostate Imaging-Reporting and Data System score; Sig = significance

**FIGURE 1.**
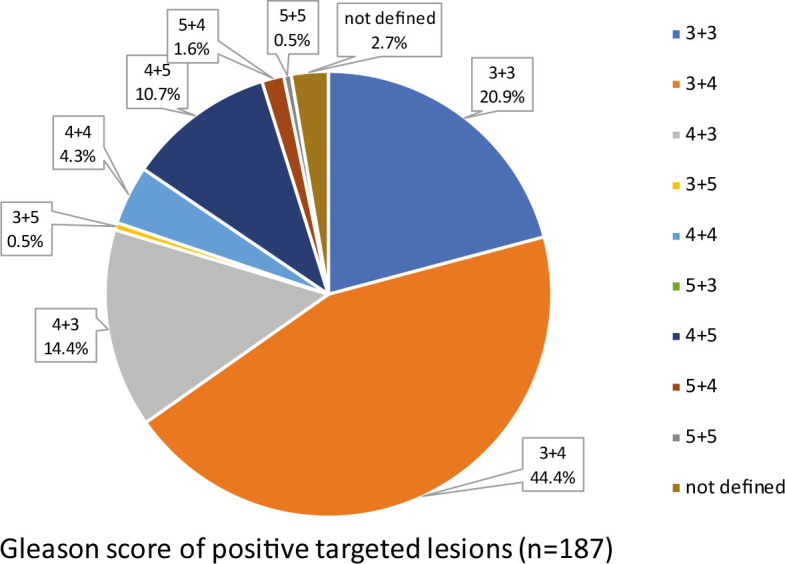
Gleason score of all positive biopsy (BX) lesions. Category »not defined« includes lesions with suspect cores for carcinoma, undefined Gleason score in invasive carcinoma and atypical small acinar proliferation (ASAP).

Our results show that 20.9% of all positive lesions had a Gleason score of 6 (International Society of Urological Pathology (ISUP) grade group 1), representing a cisPCa, while 76.4% had a Gleason score of 7 or more (ISUP grade group ≥2), representing a csPCa (Figure 1).

**FIGURE 2.**
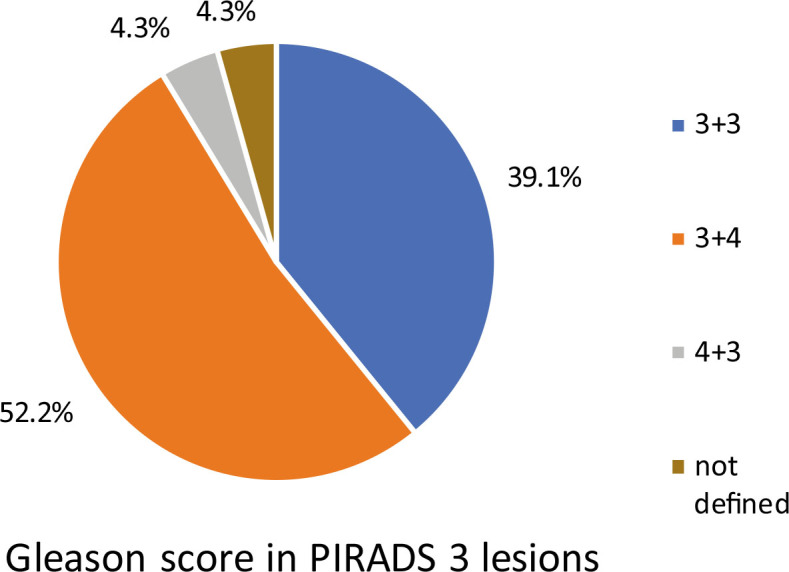
Gleason score of Prostate Imaging-Reporting and Data System (PIRADS) 3 positive biopsy (BX) lesions.

Stratifying Gleason score of lesions across PIRADS 3 category shows that 56.5% of the positive lesions had a Gleason score of 7 (Figure 2).

### Complication rate analysis

Of the 293 patients, 13 (4.4%) were found to have complications. The infectious complications that required hospitalization were: epididymitis in 1 patient (0.3%), prostatitis in 5 patients (1.7%) and urosepsis in 2 patients (0.7%). Hematuria treated on an outpatient basis was observed in 2 patients (0.7%). Minor complications immediately after BX (nausea, syncope and dizziness) occurred in 3 patients (0.9%).

## Discussion

### Patient based analysis discussion

In our study, we evaluated the performance and learning curve of the MRI-TRUS fusion BX process in the first year after its introduction in our department. Compared with our previous series from before the mpMRI era, which was based on data from 5272 patients with systematic BX (2009 to 2013) and in which 39.8% patients had a positive histopathologic result^25^, the present study showed a significantly higher proportion of patients with a positive histopathologic result in both targeted and systematic BX (53.9% and 47.9%, respectively) and overall BX (targeted plus systematic BX) (63.9%). In addition, the number of patients undergoing prostate BX has significantly decreased from an average of 1054 patients per year in 2009–2013 to 391 patients per year (293 patients with targeted and systematic BX in the study and 98 patients with systematic BX only who were not included in the study) due to mpMRI, which is now a main diagnostic method for the indication of BX in patients with elevated PSA concentration. Our present data are similar to the results of a recent study that reported a positive histopathologic result for systematic BX in 57% of patients and for general BX in 68% of patients.^26^ Although the advantage of targeted BX over systematic BX seems obvious, systematic BX adds up to 11% over targeted PCa detection rates alone, so the combination of targeted and systematic BX is still recommended.^27^ In addition, Malewski *et al*. investigated the added value of systematic BX in patients with PIRADS 5 lesions and argued that the identification of other PCa foci besides the index lesion with systemic BX could influence the treatment decision.^28^

Contrary to expectations, we did not observe significant differences in the proportions of patients with positive histopathologic result in targeted, systematic and overall BX when patients were stratified into time-based groups (2, 3, or 6 groups), which should reflect the learning curve for the overall MRI-TRUS fusion process of targeted BX in our department at the institutional level, including the process of identifying lesions described in the radiologic report and contouring them. Comparison of PCa detection rates between urologists also showed no significant differences, although the number of BX sessions varied significantly between urologists. According to studies, the learning curve of MRI-TRUS fusion in targeted BX flattens out at the individual level between 50 and 100 patients^29,30^, however, another study that followed the progress of a single novice urologist found only an increased rate of positive cores in targeted BX, but not a significantly higher proportion of patients with positive histopathologic result in the second group of 42 patients compared to the first group of 42 patients.^31^ In addition, a recent study comparing the results of MRI-TRUS fusion targeted BX between consultants and trained residents found no significant difference in PCa detection rates, duration of the procedure, pain, or complication rates of the BX procedure.^32^ One of the reasons for variable results in terms of learning curve may suggest that the technological advancement of MRI-TRUS fusion-targeted BX devices has reached a level where individual differences between operators are mitigated.

### Lesion based analysis discussion

In the second part of our study, we focused on the targeted lesion analysis. The most important variable influencing the probability of a lesion being PCa positive is the PIRADS classification, followed by prostate volume, the number of cores taken from a lesion and the total PSA concentration. The odds ratio for a positive PIRADS 4 and 5 lesion is more than 3 and 16 times higher, respectively, than for a PIRADS 3 lesion. Surprisingly, the maximum lesion diameter had no significant influence on the probability of a lesion being PCa positive. The rates of positive lesions stratified by PIRADS category in our study, with the exception of PIRADS 4, are similar to the results of a recent study in which 17%, 63%, and 88% of all lesions were positive in PIRADS 3, 4, and 5, respectively.^33^ The low rate of positive targeted BX in PIRADS 3 lesions raises the question of whether PIRADS 3 lesions should undergo BX at all. Figure 2 suggests that if PIRADS 3 lesions were excluded from BX, 56.5% PIRADS 3 lesions with csPCa would have been missed, but the absolute number of missed lesions in our study would be only 13. Schenker *et al*. have reported that although PIRADS 3 lesions have an equivocal probability of csPCa, their study found an overall PCa detection rate of only 26.8% and 14.6% for csPCa in these lesions, respectively^34^, while Nicola *et al*. have suggested that PSA density, age, and tumor volume should be considered when deciding on BX of PIRADS 3 lesions.^35^

In the present study, the proportion of csPCa-positive lesions among all targeted BX lesions was 34.7% (76.4% of 45.4% lesions), which is comparable to data from the literature.^36^ Of all positive lesions, 76.4% were csPCa, suggesting a relatively low rate of PCa overdiagnosis in our series.

### Discussion on complication rates

One of the main reasons why the EAU guidelines recommend the transperineal approach for prostate biopsy is the lower rate of postprocedural infectious complications, even though the transperineal approach is often associated with significant logistical problems^3^, which are particularly problematic for high-volume centers (e.g., the need for general anesthesia in the operating room and the longer duration of the procedure). Our data show that the cumulative hospitalization rate due to infectious complications with the transrectal approach was 2.7%, but most of the patients received only one dose of antibiotic prophylaxis with phosphomycin the evening before BX during the study period, which was adjusted the following year with an additional dose of phosphomycin 24 hours after the procedure. The sepsis rate was 0.7% in the first year, which is significantly lower than in a recent meta-analysis of transrectal procedures, in which the subgroup of patients with antibiotic prophylaxis had a sepsis rate of 1.7%, and comparable to the subgroup of patients who received rectal disinfection with povidone-iodine before BX in addition to antibiotic prophylaxis (0.6%). Furthermore, our sepsis rate was significantly lower compared to a UK national study of 73630 patients comparing the transperineal (1.03% sepsis rate) to the transrectal (1.35% sepsis rate) approach^37^, and comparable to the sepsis rates (0.7%) cited by Cheng *et al*. who also estimate the cumulative rate of infectious conditions to be 2%.^37^ In addition, the ProBE-PC clinical trial compared infectious and noninfectious complications in 351 and 367 patients undergoing transrectal and transperineal prostate BX, respectively. Cumulative infectious events occurred in 2.6% and 2.7% of participants for transrectal and transperineal prostate BX, respectively, while none of the participants in either group developed sepsis.^38^

Besides retrospecitve nature, the main limitation of the present study is the relatively large number of participating urologists and radiologists from different radiology centers with different MRI equipment in Slovenia, which resulted in considerable heterogeneity in the reporting of mpMRI and performing MRI-TRUS fusion BX and might also affect our results and conclusions on learning curve. On the other hand, this short-coming is mitigated to some extent by the large number of patients in the study and the fact that prostate contouring and supervision of BX process was performed by a small number of experienced urologists. In addition, the results reflect the real-life circumstances in high-volume centers where it is rarely possible to ensure strictly regulated research conditions. Patel *et al*. analyzed reports from 10 radiologists performing mpMRI and 5 urologists performing MRI-TRUS fusion BX PCa in 865 patients to estimate individual variability in overall and csPCa detection rates and found significant variability among radiologists but not among urologists, although both rates improved over time. They concluded that improving the quality of mpMRI PIRADS reporting is a key area to focus on.^36^

## Conclusions

The introduction of MRI-TRUS fusion to targeted BX significantly improves the overall rate of PCa detection compared to systematic BX alone, however systematic BX should still be performed during targeted BX session in the contemporary clinical practice. Due to the simplified technical aspects of the BX procedure, no steep learning curve was observed among our urologists. The proportion of lesions with cisPCa was low, limiting the overdiagnosis of PCa. The rate of infectious complications was acceptable and not inferior to published data on transrectal and transperineal BX.

## AI disclosure

Statement: During the preparation of this paper, the authors used InstaText for Word tool to improve English language in the manuscript. After using this tool, the authors have reviewed and edited the content as required and take full responsibility for the content of the publication.

## References

[1] Culp MB, Soerjomataram I, Efstathiou JA, Bray F, Jemal A (2020). Recent global patterns in prostate cancer incidence and mortality rates. Eur Urol.

[2] Zadnik V, Gašljević G, Hočevar M, Jarm K, Pompe-Kirn V, Strojan P (2022). Cancer in Slovenia 2019.

[3] Cornford P, Tilki D, van den Bergh RCN, Briers E, Eberli D, De Meerleer M (2024). Prostate cancer. EAU guidelines 2024.

[4] Stamey TA, Yang N, Hay AR, McNeal JE, Freiha FS, Redwine E (1987). Prostate-specific antigen as a serum marker for adenocarcinoma of the prostate. N Engl J Med.

[5] Sokoll LJ, Chan DW, Mikolajczyk SD, Rittenhouse HG, Evans CL, Linton HJ (2003). Proenzyme psa for the early detection of prostate cancer in the 2.5–4.0 ng/ml total psa range: preliminary analysis. Urology.

[6] Hedelin H, Johansson N, Stroberg P (2005). Relationship between benign prostatic hyperplasia and lower urinary tract symptoms and correlation between prostate volume and serum prostate-specific antigen in clinical routine. Scand J Urol Nephrol.

[7] Chambo RC, Tsuji FH, de Oliveira Lima F, Yamamoto HA, de Jesus CM (2014). What is the ideal core number for ultrasound-guided prostate biopsy?. Korean J Urol.

[8] Walz J, Graefen M, Chun FK, Erbersdobler A, Haese A, Steuber T (2006). High incidence of prostate cancer detected by saturation biopsy after previous negative biopsy series. Eur Urol.

[9] Bell KJ, Del Mar C, Wright G, Dickinson J, Glasziou P (2015). Prevalence of incidental prostate cancer: a systematic review of autopsy studies. Int J Cancer.

[10] Jansen FH, van Schaik RH, Kurstjens J, Horninger W, Klocker H, Bektic J (2010). Prostate-specific antigen (PSA) isoform p2PSA in combination with total PSA and free PSA improves diagnostic accuracy in prostate cancer detection. Eur Urol.

[11] Loeb S, Catalona WJ (2014). The Prostate Health Index: a new test for the detection of prostate cancer. Ther Adv Urol.

[12] Deras IL, Aubin SM, Blase A, Day JR, Koo S, Partin AW (2008). PCA3: a molecular urine assay for predicting prostate biopsy outcome. J Urol.

[13] Van Neste L, Hendriks RJ, Dijkstra S, Trooskens G, Cornel EB, Jannink SA (2016). Detection of high-grade prostate cancer using a urinary molecular biomarker-based risk score. Eur Urol.

[14] Futterer JJ (2017). Multiparametric MRI in the detection of clinically significant prostate cancer. Korean J Radiol.

[15] Drost FH, Osses DF, Nieboer D, Steyerberg EW, Bangma CH, Roobol MJ (2019). Prostate MRI, with or without MRI-targeted biopsy, and systematic biopsy for detecting prostate cancer. Cochrane Database Syst Rev.

[16] Bratan F, Niaf E, Melodelima C, Chesnais AL, Souchon R, Mege-Lechevallier F (2013). Influence of imaging and histological factors on prostate cancer detection and localisation on multiparametric MRI: a prospective study. Eur Radiol.

[17] Futterer JJ, Briganti A, De Visschere P, Emberton M, Giannarini G, Kirkham A (2015). Can clinically significant prostate cancer be detected with multiparametric magnetic resonance imaging? A systematic review of the literature. Eur Urol.

[18] Weinreb JC, Barentsz JO, Choyke PL, Cornud F, Haider MA, Macura KJ (2016). PI-RADS Prostate Imaging - Reporting and Data System: 2015, Version 2. Eur Urol.

[19] Xu G, Li JH, Xiang LH, Yang B, Chen YC, Sun YK (2023). Transrectal ultrasound examination of prostate cancer guided by fusion imaging of multiparametric MRI and TRUS: avoiding unnecessary mpMRI-guided targeted biopsy. Asian J Androl.

[20] Littrup PJ, Bailey SE (2000). Prostate cancer: the role of transrectal ultrasound and its impact on cancer detection and management. Radiol Clin North Am.

[21] Wegelin O, Exterkate L, van der Leest M, Kummer JA, Vreuls W, de Bruin PC (2019). The FUTURE Trial: a multicenter randomised controlled trial on target biopsy techniques based on magnetic resonance imaging in the diagnosis of prostate cancer in patients with prior negative biopsies. Eur Urol.

[22] Simmons LAM, Kanthabalan A, Arya M, Briggs T, Barratt D, Charman SC (2018). Accuracy of transperineal targeted prostate biopsies, visual estimation and image fusion in men needing repeat biopsy in the PICTURE Trial. J Urol.

[23] Wegelin O, van Melick HHE, Hooft L, Bosch J, Reitsma HB, Barentsz JO (2017). Comparing three different techniques for magnetic resonance imaging-targeted prostate biopsies: a systematic review of in-bore versus magnetic resonance imaging-transrectal ultrasound fusion versus cognitive registration. Is there a preferred technique?. Eur Urol.

[24] Watts KL, Frechette L, Muller B, Ilinksy D, Kovac E, Sankin A (2020). Systematic review and meta-analysis comparing cognitive vs. image-guided fusion prostate biopsy for the detection of prostate cancer. Urol Oncol.

[25] Smrkolj T (2015). [Transrectal ultrasound and needle biopsy of the prostate]. [Slovenian]. Zdrav Vestn.

[26] Ortner G, Mavridis C, Fritz V, Schachtner J, Mamoulakis C, Nagele U (2024). The added value of MRI-based targeted biopsy in biopsy-naive patients: a propensity-score matched comparison. J Clin Med.

[27] Connor MJ, Miah S, Jayadevan R, Khoo CC, Eldred-Evans D, Shah T (2020). Value of systematic sampling in an mpMRI targeted prostate biopsy strategy. Transl Androl Urol.

[28] Malewski W, Milecki T, Szemplinski S, Tayara O, Kuncman L, Kryst P (2023). Prostate biopsy in the case of PIRADS 5 - is systematic biopsy mandatory?. J Clin Med.

[29] Kasabwala K, Patel N, Cricco-Lizza E, Shimpi AA, Weng S, Buchmann RM (2019). The learning curve for magnetic resonance imaging/ultrasound fusion-guided prostate biopsy. Eur Urol Oncol.

[30] Xu L, Ye NY, Lee A, Chopra J, Naslund M, Wong-You-Cheong J (2023). Learning curve for magnetic resonance imaging/ultrasound fusion prostate biopsy in detecting prostate cancer using cumulative sum analysis. Curr Urol.

[31] Mager R, Brandt MP, Borgmann H, Gust KM, Haferkamp A, Kurosch M (2017). From novice to expert: analyzing the learning curve for MRI-transrectal ultrasonography fusion-guided transrectal prostate biopsy. Int Urol Nephrol.

[32] Turchi B, Lombardo R, Franco A, Tema G, Nacchia A, Cicione A (2024). Residents and consultants have equal outcomes when performing transrectal fusion biopsies: a randomized clinical trial. Curr Oncol.

[33] Rodriguez-Cabello MA, Mendez-Rubio S, Sanz-Miguelanez JL, Moraga-Sanz A, Aullo-Gonzalez C, Platas-Sancho A (2023). Prevalence and grade of malignancy differences with respect to the area of involvement in multiparametric resonance imaging of the prostate in the diagnosis of prostate cancer using the PI-RADS version 2 classification. World J Urol.

[34] Schlenker B, Apfelbeck M, Armbruster M, Chaloupka M, Stief CG, Clevert DA (2019). Comparison of PIRADS 3 lesions with histopathological findings after MRI-fusion targeted biopsy of the prostate in a real world-setting. Clin Hemorheol Microcirc.

[35] Nicola R, Bittencourt LK (2023). PIRADS 3 lesions: a critical review and discussion of how to improve management. Abdom Radiol (NY).

[36] Patel HD, Halgrimson WR, Sweigert SE, Shea SM, Turk TMT, Quek ML (2024). Variability in prostate cancer detection among radiologists and urologists using MRI fusion biopsy. BJUI Compass.

[37] Berry B, Parry MG, Sujenthiran A, Nossiter J, Cowling TE, Aggarwal A (2020). Comparison of complications after transrectal and transperineal prostate biopsy: a national population-based study. BJU Int.

[38] Mian BM, Feustel PJ, Aziz A, Kaufman RP, Bernstein A, Avulova S (2024). Complications following transrectal and transperineal prostate biopsy: results of the ProBE-PC Randomized Clinical Trial. J Urol.

